# 3D Printed Pellets (Miniprintlets): A Novel, Multi-Drug, Controlled Release Platform Technology

**DOI:** 10.3390/pharmaceutics11040148

**Published:** 2019-03-29

**Authors:** Atheer Awad, Fabrizio Fina, Sarah J. Trenfield, Pavanesh Patel, Alvaro Goyanes, Simon Gaisford, Abdul W. Basit

**Affiliations:** 1 Department of Pharmaceutics, UCL School of Pharmacy, University College London, 29-39 Brunswick Square, London WC1N 1AX, UK; atheer.awad.15@ucl.ac.uk (A.A.); fabrizio.fina.14@ucl.ac.uk (F.F.); sarah.trenfield.16@ucl.ac.uk (S.J.T.); pavanesh.patel.14@ucl.ac.uk (P.P.); s.gaisford@ucl.ac.uk (S.G.); 2 FabRx Ltd., 3 Romney Road, Ashford, Kent TN24 0RW, UK; 3 Departamento de Farmacología, Farmacia y Tecnología Farmacéutica, R + D Pharma Group (GI-1645), Universidade de Santiago de Compostela, 15782 Santiago de Compostela, Spain

**Keywords:** three dimensional printing, additive manufacturing, 3D printed drug products, printlets, personalised medicines, personalized pharmaceuticals, multiple units, spheroids, beads, acetaminophen

## Abstract

Selective laser sintering (SLS) is a single-step three-dimensional printing (3DP) process that can be leveraged to engineer a wide array of drug delivery systems. The aim of this work was to utilise SLS 3DP, for the first time, to produce small oral dosage forms with modified release properties. As such, paracetamol-loaded 3D printed multiparticulates, termed miniprintlets, were fabricated in 1 mm and 2 mm diameters. Despite their large surface area compared with a conventional monolithic tablet, the ethyl cellulose-based miniprintlets exhibited prolonged drug release patterns. The possibility of producing miniprintlets combining two drugs, namely paracetamol and ibuprofen, was also investigated. By varying the polymer, the dual miniprintlets were programmed to achieve customised drug release patterns, whereby one drug was released immediately from a Kollicoat Instant Release matrix, whilst the effect of the second drug was sustained over an extended time span using ethyl cellulose. Herein, this work has highlighted the versatility of SLS 3DP to fabricate small and intricate formulations containing multiple active pharmaceutical ingredients with distinct release properties.

## 1. Introduction

Three-dimensional printing (3DP) is a revolutionary additive manufacturing technology that can transform 3D designs into real objects by sequential layering [1]. Its applications are broad, ranging from aviation and automobiles to human organs and implants [2,3]. Unlike conventional production methods, 3DP has the capability to accurately distribute materials, facilitating the production of medications with individualised doses [4,5] and polypills with multiple active pharmaceutical ingredients (APIs), wherein each drug can be placed in a different layer [6] or compartment [7]. In addition, this technology can create highly precise dosage forms with varying shapes [8] and sizes [9,10], enabling the local delivery of drugs to specified organs [11,12,13] and offering versatile drug release modes [14,15]. Within healthcare, 3DP is forecast to transition medication production from centralised facilities to decentralised spaces, such as clinics, hospitals and local pharmacies [16,17]. More specifically, this technique could be implemented as a digitised production tool for the remote design, development and dispensing of bespoke medications optimised to each patient’s needs [18,19].

Selective laser sintering (SLS) is a rapid 3DP process that offers a one-step, solvent-free method for production [20,21]. This technology utilises a laser beam to selectively bind powdered materials together to produce 3D objects in a layered manner [22]. To date, SLS has shown great flexibility, enabling it to fabricate a wide array of dosage forms, with different shapes [23] and release characteristics ranging from orally-disintegrating tablets [24] to immediate and modified release dosage forms [25]. The high precision of the laser permits the printing of very detailed lattice structures with controllable internal architectures, which are otherwise impossible to produce using conventional production methods [26,27,28]. The high resolution of the SLS 3DP system may render this technology suitable for the preparation of multiparticulate drug delivery systems.

Compared with single-unit dosage forms, multiparticulate systems offer a more attractive means for dosing flexibility, with additional therapeutic benefits to the patients [29]. As an example, multiple-unit dosage forms can be divided into desired doses without necessitating alteration of the formulation or manufacturing process. They also have a more reproducible and predictable journey through the gastrointestinal tract. For example, the spreading of multi-unit medicines means that the formulation will be more exposed to fluids during its transit; thus, multiparticulates are free to disperse in the gastrointestinal tract, which can maximise drug release and drug absorption. Moreover, in the presence of food, the gastric emptying of these systems is considered to be more uniform when compared to single-oral dosage units [30]. Furthermore, given that recent studies have shown that some dosage forms, having a similar size to enteric coated size 9 capsules, do not actually empty from the stomach of small rodents [31], the use of multiparticulate systems provides a clear advantage over other pharmaceutical products. As such, these dosage forms are suited for administration to various animal species, where they can be printed in dimensions and doses personalised to the model, making them applicable throughout the whole drug development pathway. 

An important challenge in formulating small dosage forms lies in the difficulty of controlling the drug release from the polymeric matrices and the low drug loading potential in comparison with conventional manufacturing processes [32]. A potential benefit of the SLS technology lies in its laser sintering process, which fuses the drug and polymer particles together, producing a strong coherence between the particles and sustaining the drug release from the molten matrix. Moreover, the high resolution of the laser beam enables the printing of very small and detailed units. In fact, the additive nature of the process offers the advantage of higher control over the composition and content distribution within the printed formulations, which in turn may render this technology highly accurate and reproducible, enabling it to produce multi-drug iterations with distinct and uniform dosing. The discrete separation of the APIs resolves problems associated with compatibility and physical interaction, which are a major setback in most multi-drug formulations. More notably, the ease of preparation of the SLS feedstock makes the overall process quick, user-friendly and economical. 

Hence, the aim of this study was to investigate the suitability of SLS 3DP for manufacturing small dosage forms with modified release properties, herein termed miniprintlets. Paracetamol-loaded miniprintlets with sustained drug release kinetics were created in two different sizes (1 mm and 2 mm in diameter), to evaluate the effect of changing the size on in vitro dissolution. SLS was further used for the dual printing of miniprintlets with two rate-controlling systems incorporating two model drugs, paracetamol and ibuprofen. To our knowledge, this work is the first to report the engineering of such small and intricate 3D printed formulations containing multiple APIs with distinct release properties. 

## 2. Materials and Methods 

Paracetamol USP grade (MW 151.16 g/mol, solubility in water at 37 °C: 21.80 g/L) and ibuprofen (sodium salt; MW 228.26 g/mol, solubility in water: 100 g/L) (both from Sigma-Aldrich, Poole, UK) were used as model drugs. Ethyl cellulose N7 was obtained from Ashland, Schaffhausen, Switzerland and Kollicoat Instant Release (IR) was obtained from BASF, Ludwigshafen, Germany. Candurin Gold Sheen was purchased from Merck, Darmstadt, Germany. The salts for preparing the buffer dissolution media were purchased from VWR International Ltd., Leicestershire, UK.

### 2.1. Printing Process

**Figure 1:**
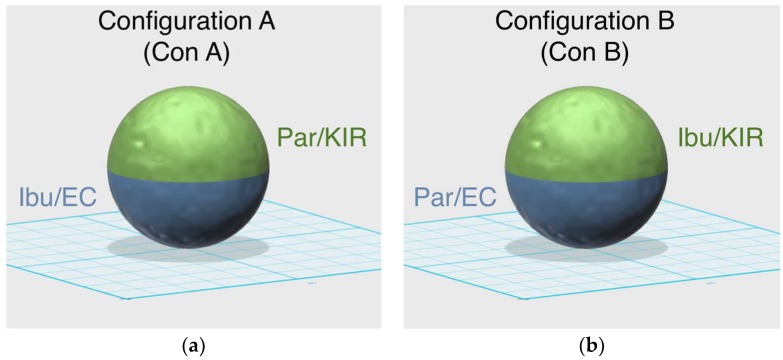
Schematic representation of the compositions of (**a**) configuration A and (**b**) configuration B of the dual miniprintlets.

All the powders were sieved using a 150 μm sieve prior to their use to permit a better flow of the powder particles in the chamber, resulting in a better printing procedure [33]. For all the formulations, 100 g of a mixture of drug and excipients were blended using a mortar and pestle (Table 1). 3% Candurin Gold Sheen was added to the formulations to enhance energy absorption from the laser and aid printability. The single miniprintlets were loaded with 5% paracetamol, whereas the dual miniprintlets contained 6.5% paracetamol and 3.5% ibuprofen, which is the ratio at which combinations containing these drugs tend to be at. Powder mixtures were transferred to a desktop SLS printer (Sintratec Kit, AG, Brugg, Switzerland) to fabricate the oral dosage formulations. 123D Design (Version 14.2.2, Autodesk Inc., San Rafael, CA, USA) was used to design the templates of the spherical miniprintlets (1 mm and 2 mm in diameter). The 3D models were exported as a stereolithography (.stl) file into the 3D printer Sintratec central software (Version 1.1.13, Sintratec, AG, Brugg, Switzerland). The dual miniprintlets consisted of two distinct regions, wherein the one region had immediate release properties and the other region exhibited sustained release (Figure 1). As such, to prevent the mixing of the two powders and permit precise control over the content of each region, the reservoir platform was kept empty and instead, the powders were manually added to the building platform before the start of each layer. From our observation, the manual addition of the powder slightly lengthened the printing process, as the freshly added layer required additional heating prior to the start of the printing. The dual miniprintlets were fabricated in two different configurations; in configuration A (Con A), paracetamol was mixed with Kollicoat IR (Par/KIR region) and ibuprofen was with ethyl cellulose (Ibu/EC region). Comparatively, in configuration B (Con B), the positions of the drugs were switched and paracetamol was mixed with ethyl cellulose (Par/EC region), whilst ibuprofen was with Kollicoat IR (Ibu/KIR region).

Powder in the reservoir platform (150 mm × 30 mm × 150 mm) of the printer was moved to the building platform (150 mm × 30 mm × 150 mm) by a sled, producing a flat and homogeneously distributed layer of powder. The chamber and surface printing temperatures for all the miniprintlets were 100 °C and 120 °C, respectively. A 2.3 W blue diode laser (445 nm), with a scanning speed of 50 mm/s, was activated to sinter the powder on to the building platform based on the STL file. At this point, the reservoir platform moved up, the building platform moved down, and the sled distributed a thin layer of powder on top of the previous layer. This process was repeated layer-by-layer until the object was completed. The powder was then removed from the chamber and sieved using a 710 μm sieve to recover the miniprintlets. For each batch, 100 miniprintlets were printed at a time. Before the start of printing, the printer required ~10 min to warm up the platforms; the time was, however, reduced to 2–3 min in successive printing jobs, as the printer was able to maintain the heat within its chamber. 

### 2.2. Thermal Analysis

Differential scanning calorimetry (DSC) was used to characterise the powders and the drug-loaded miniprintlets. DSC measurements were performed with a Q2000 DSC (TA instruments—Waters LLC, New Castle, DE, USA) at a temperature range of 0 °C to 200 °C and a heating rate of 10 °C/min. Calibration for cell constant and enthalpy was performed with indium (T_m_ = 156.6 °C, ∆H_f_ = 28.71 J/g), according to the manufacturer’s instructions. Nitrogen was used as a purge gas with a flow rate of 50 mL/min for all the experiments. Data were collected with TA Advantage software for Q series (Version 2.8.394) and analysed using TA Instruments Universal Analysis 2000 (TA instruments—Waters LLC, New Castle, DE, USA). All melting temperatures are reported as extrapolated onset unless otherwise stated. TA aluminium pans and lids (Tzero) were used with an average sample mass of 3–5 mg. 

For thermogravimetric analysis (TGA), average samples of 8–10 mg of raw drugs, polymers and powder mixtures were heated at a temperature range of 50 °C to 500 °C and a heating rate of 10 °C/min in open aluminium pans using a Discovery TGA (TA instruments—Waters LLC, New Castle, DE, USA). Nitrogen, at a flow rate of 25 mL/min, was used as a purge gas. Data were collected and analysed using TA Instruments Trios software (Version 4.5.0.5), where the percentage mass loss with respect to temperature was calculated.

### 2.3. X-ray Powder Diffraction (XRPD)

Discs of 23 mm diameter × 1 mm height made from the mixtures of drugs and excipients were 3D printed and analysed. Samples of pure drugs, polymers and powder mixtures were also analysed. The X-ray powder diffraction patterns were obtained in a Rigaku MiniFlex 600 (Rigaku, Wilmington, MA, USA) using a Cu Kα X-ray source (λ = 1.5418 Å). The intensity and voltage applied were 15 mA and 40 kV, respectively. The angular range of data acquisition was 3–40° 2θ, with a stepwise size of 0.02° at a speed of 2°/min. 

### 2.4. Characterisation of the Miniprintlets

#### 2.4.1. Determination of the Miniprintlets Morphology

The diameter of the miniprintlets was measured using a digital caliper. For the weight and diameter measurements, 5 and 10 miniprintlets were used, respectively, from each formulation. For the diameter measurements, the average of the longest and shortest distances was used for each miniprintlet.

#### 2.4.2. Scanning Electron Microscopy (SEM) 

Surface images of the 2 mm miniprintlets were taken with a scanning electron microscope (SEM, JSM-840A Scanning Microscope, JEOL GmbH, Freising, Germany). All samples for SEM testing were coated with carbon (~30–40 nm). 

#### 2.4.3. X-ray Micro Computed Tomography (Micro-CT)

A high-resolution X-ray micro computed tomography (Micro-CT) scanner (SkyScan1172, Bruker-microCT, Kontich, Belgium) was used to three-dimensionally visualise the internal structure and calculate the density of the miniprintlets. In this study, 2 mm dual miniprintlets were scanned with a resolution of 2000 × 1048 pixels. 3D imaging was performed by rotating the object through 180° with steps of 0.4° and four images were recorded for each of those. Image reconstruction was performed using NRecon software (Version 1.7.0.4, Bruker-microCT) and 3D model rendering and viewing were performed using the associate program CT-Volume software (Version 2.3.2.0). The collected data were analysed using Analyzer (Version 1.16.4.1), where maps of different colours were used to represent the density of the miniprintlets. 

#### 2.4.4. Determination of Drug Content

In total, 20–25 mg of miniprintlets from each size were placed in separate volumetric flasks containing 10 mL of methanol. For the dual miniprintlets, single miniprintlets consisting of the individualised composition of each region were printed using the same parameters and were used for the drug content testing. Miniprintlets consisting of Kollicoat IR were dissolved in 10 mL of water, whereas the miniprintlets consisting of ethyl cellulose were dissolved in 10 mL of methanol. Samples of solution were then filtered through 0.22 µm filters (Millipore Ltd., Cork, Ireland) and the drug concentrations were determined with high-performance liquid chromatography (HPLC) (Hewlett Packard 1050 Series HPLC system, Agilent Technologies, Cheshire, UK). The validated HPLC assay entailed injecting 20 µL samples for analysis, using a mobile phase with a gradient elution system of (A) acetonitrile and (B) 0.1% formic acid in distilled water, through an Eclipse plus C18 3.5 µm column, 4.6 × 100 mm (Zorbax, Agilent technologies, Cheshire, UK), maintained at 40 °C. The mobile phase was pumped at a flow rate of 1 mL/min under the following gradient program: 0−2 min, 15% A; 2–8 min, 15–55% A; 8–25 min, 55% A; 25–26 min, 55–15% A. The eluents were screened at a wavelength of 230 nm, where the retention times for paracetamol and ibuprofen were 2.57 min and 14.36 min, respectively. The tests were done in triplicate.

### 2.5. In-Vitro Dissolution Testing 

Drug dissolution profiles for the miniprintlets were obtained with a USP-II apparatus (Pharmatest PTWS 100, Haiburg, Germany): (1) 250 mg of miniprintlets were placed within in-house sinkers and dissolved in 750 mL of 0.1 M HCl for 2 h to simulate gastric residence time, and (2) were then transferred into 950 mL of modified Hanks (mHanks) bicarbonate physiological medium for 35 min (pH 5.6 to 7); (3) and in modified Krebs buffer (1000 mL) (pH 7 to 7.4 and then to 6.5). The modified Hanks buffer-based dissolution medium [34] (136.9 mM NaCl, 5.37 mM KCl, 0.812 mM MgSO_4_.7H_2_O, 1.26 mM CaCl_2_, 0.337 mM Na_2_HPO_4_.2H_2_O, 0.441 mM KH_2_PO_4_, 4.17 mM NaHCO_3_) forms an in-situ modified Kreb’s buffer [35] through the addition of 50 mL of pre-Krebs solution (400.7 mM NaHCO_3_ and 6.9 mM KH_2_PO_4_) to each dissolution vessel.

The miniprintlets were tested in the small intestinal environment for 3.5 h (pH 5.6 to 7.4) [36]. The medium is primarily a bicarbonate buffer in which bicarbonate (HCO_3_^−^) and carbonic acid (H_2_CO_3_) co-exist in equilibrium, along with CO_2_ (aq) resulting from dissociation of the carbonic acid. The pH of the buffer is controlled by an Auto pH System^TM^ [37,38], which consists of a pH probe connected to a source of carbon dioxide gas (pH-reducing gas), as well as to a supply of helium (pH-increasing gas), controlled by a control unit. The control unit is able to provide a dynamically adjustable pH during testing (dynamic conditions) and to maintain a uniform pH value over the otherwise unstable bicarbonate buffer pH. The paddle speed of the USP-II was fixed at 50 rpm and the tests were conducted at 37 ± 0.5 °C (n = 3). The percentage of drug released from the miniprintlets was determined using HPLC, as described in Section 2.4.4.

## 3. Results and Discussion

SLS 3DP was successfully utilised to create miniprintlets in two different diameters, 1 mm and 2 mm. Paracetamol was employed as the model drug and ethyl cellulose was employed as the main polymer matrix. A laser scanning speed of 50 mm/s was selected because it was found to provide enough energy for the effective bonding of consecutive printing layers, while maintaining the desired shape and dimensions of the miniprintlets. The time needed to print one batch of 100 miniprintlets of 1 mm was ~2 min, whereas the time need to print one batch of 2 mm miniprintlets was ~2 min and 40 s. 

Dual miniprintlets for multi-drug therapy were also fabricated, incorporating paracetamol and ibuprofen in different layers. Like the single miniprintlets, the dual miniprintlets were printed in two different sizes, 1 mm and 2 mm. The dual miniprintlets were prepared in two different configurations, wherein one drug was dispersed in Kollicoat IR, a polyvinyl alcohol/polyethylene glycol graft copolymer with immediate release characteristics, and the other drug was dispersed in ethyl cellulose. Paracetamol and ibuprofen were selected as model drugs since previous studies have shown that the combination of both drugs has a greater synergistic efficacy compared with their individual use [39,40]. The time needed to print one batch of 100 dual miniprintlets of 1 mm was ~2 min and 30 s, whereas for 2 mm miniprintlets, it was ~3 min and 40 s. The time is slightly higher than for the single miniprintlets due to the manual addition of the powders, which required additional surface heating.

Despite their small sizes, all the miniprintlets showed high uniformity in weight and diameter (Table 2). Generally, the 1 mm miniprintlets displayed a higher precision in weight when compared to the 2 mm miniprintlets. In terms of diameter, the dual miniprintlets appeared to have more precise values, wherein Con B has been shown to be more accurate when compared to theoretical diameter measurements. The 1 mm single miniprintlets, on the other hand, had the least precise and accurate readings. 

**Figure 2:**
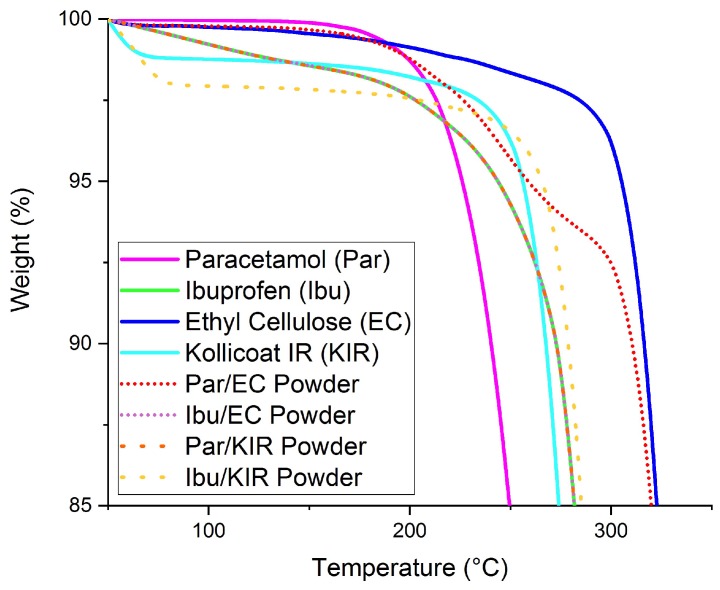
Thermogravimetric analysis (TGA) results of the raw drugs, polymers and powders prior to printing.

HPLC analysis showed that the drug content values were in agreement with the theoretical drug loadings in all the miniprintlets, confirming that no significant drug loss occurred during the printing process (Table 2), thus confirming the high accuracy and reproducibility of the SLS process. TGA data of the drugs, polymers and powder mixtures predicted that all the components would remain stable and no degradation of the drugs and excipients was likely to occur at the printing temperatures (≤120 °C) (Figure 2).

**Figure 3:**
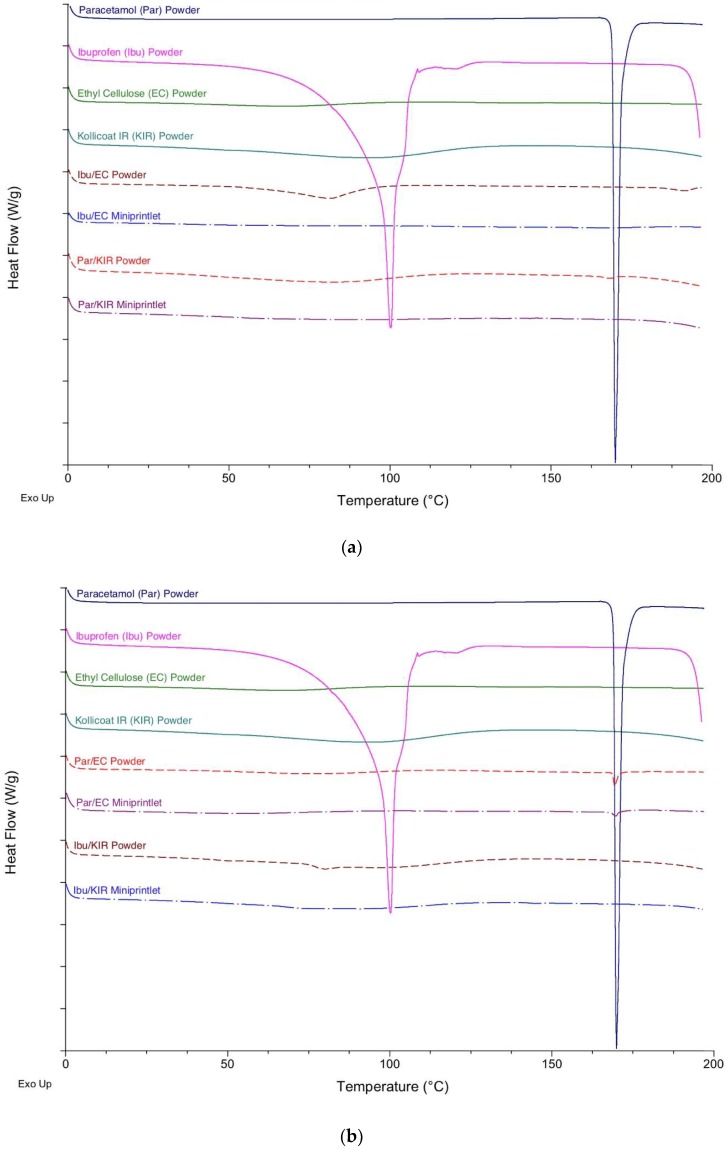
Differential scanning calorimetry (DSC) thermograms of the pure drugs, polymers and powder mixture prior to printing and the miniprintlets used in (**a**) Con A and (**b**) Con B of the dual miniprintlets.

**Figure 4:**
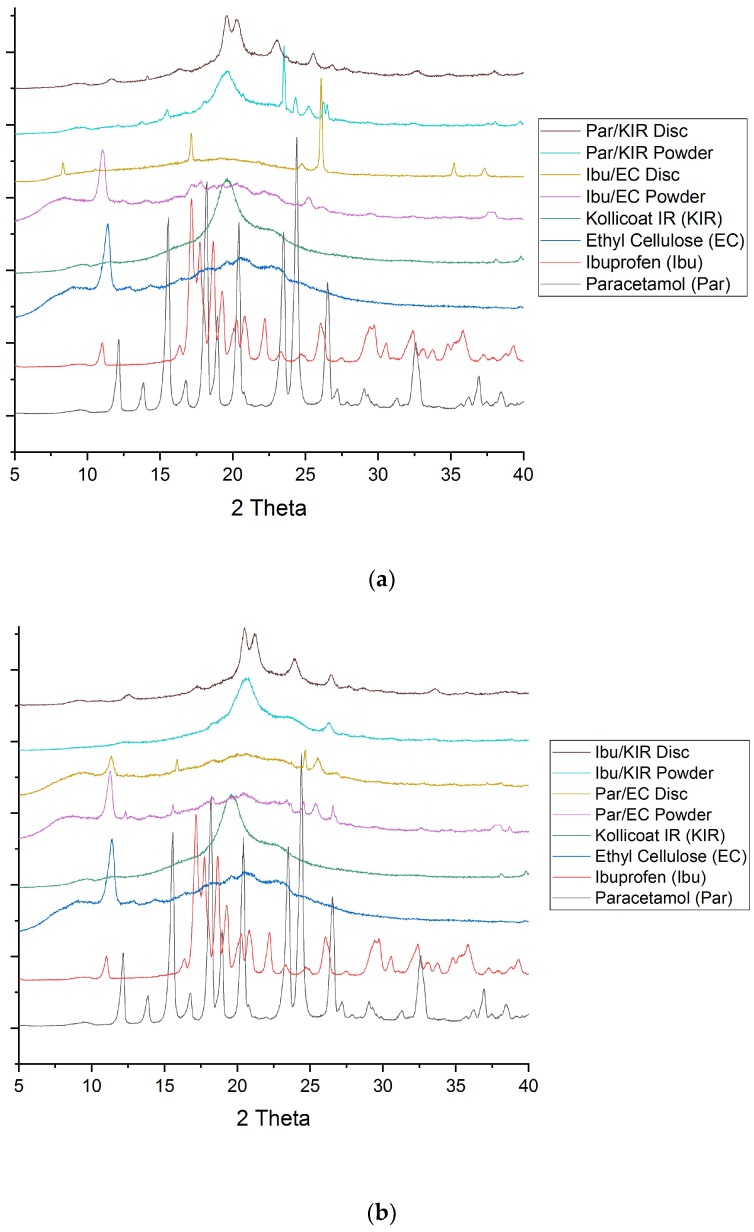
X-ray diffractograms of the drugs-excipients prior to printing and the 3D printed discs used in (**a**) Con A and (**b**) Con B of the dual miniprintlets.

DSC and XRPD analysis of the drug, polymers and powder mixtures prior to printing, and of the miniprintlets, were performed to determine the physical state of the drugs and the degree of their incorporation within the polymers (Figure 3 and Figure 4). Before printing, the DSC data showed that the raw paracetamol powder exhibited a melting endotherm at approximately 168 °C, indicative of form I [41]. The raw ibuprofen powder exhibited a broad endotherm at 100 °C, indicative of dehydration, and a sharp melting endotherm at approximately 200 °C, indicative of a racemic conglomerate, which melts at 199 °C [42]. The DSC data of the Par/EC miniprintlets showed a small melting endotherm at approximately 168 °C, demonstrating that paracetamol still exists in its crystalline form. The Par/KIR, Ibu/EC and Ibu/KIR miniprintlets, on the other hand, showed no evidence of melting endotherms, indicating that the drugs are either molecularly dispersed within the polymers or dissolved within the polymers as the temperature increases during the DSC process. Corroborating with the results obtained by DSC, the X-ray diffractograms of the Par/EC discs demonstrated that paracetamol was partially crystalline in the miniprintlets (Figure 4b). For Par/KIR, paracetamol showed less crystalline peaks in the printed discs, indicating that it had been converted to an amorphous state (Figure 4a). Interestingly, the Par/KIR and Ibu/KIR discs showed an increase in crystalline peaks, attributed to Kollicoat IR, which could be due to its exposure to heat during the sintering process [43]. 

**Figure 5:**
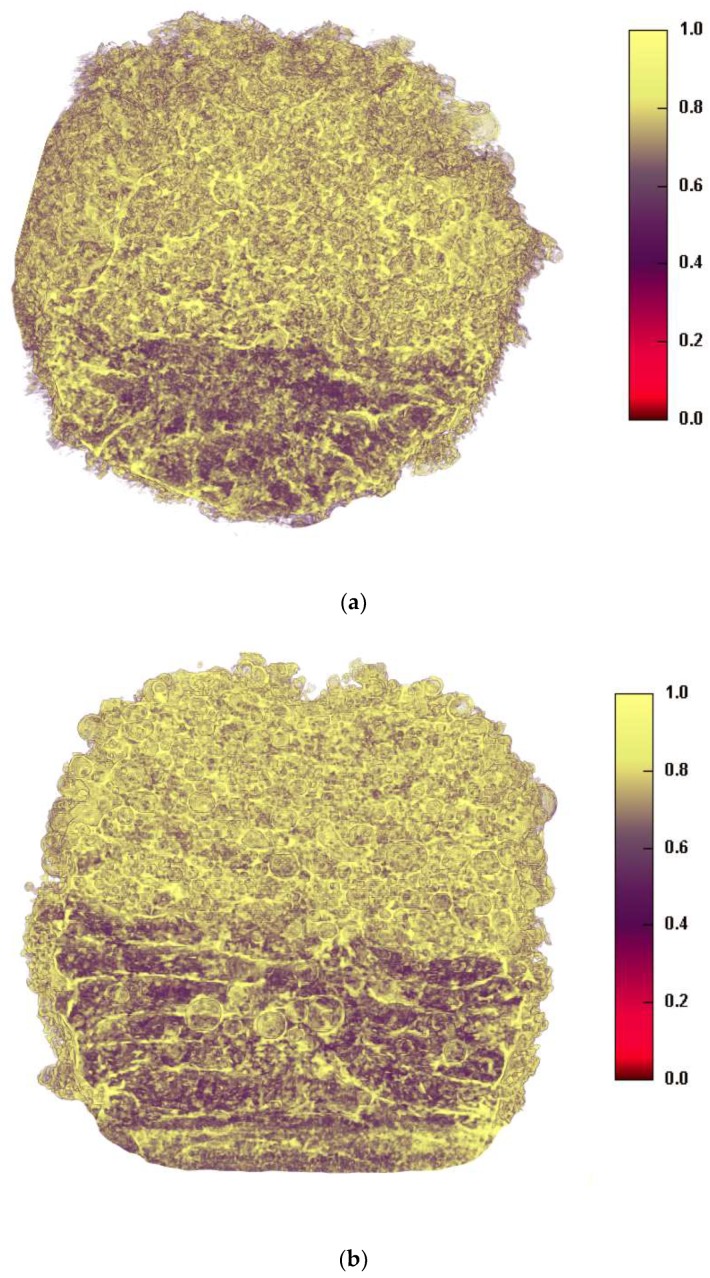
Cross-sectional x-ray micro computed tomography (CT) images of (**a**) Con A and (**b**) Con B of the 2 mm dual miniprintlets. The yellow regions represent the Kollicoat Instant Release (KIR) regions, whereas the dark purple regions are the ethyl cellulose (EC) regions. The scale bar is representative of density.

**Figure 6:**
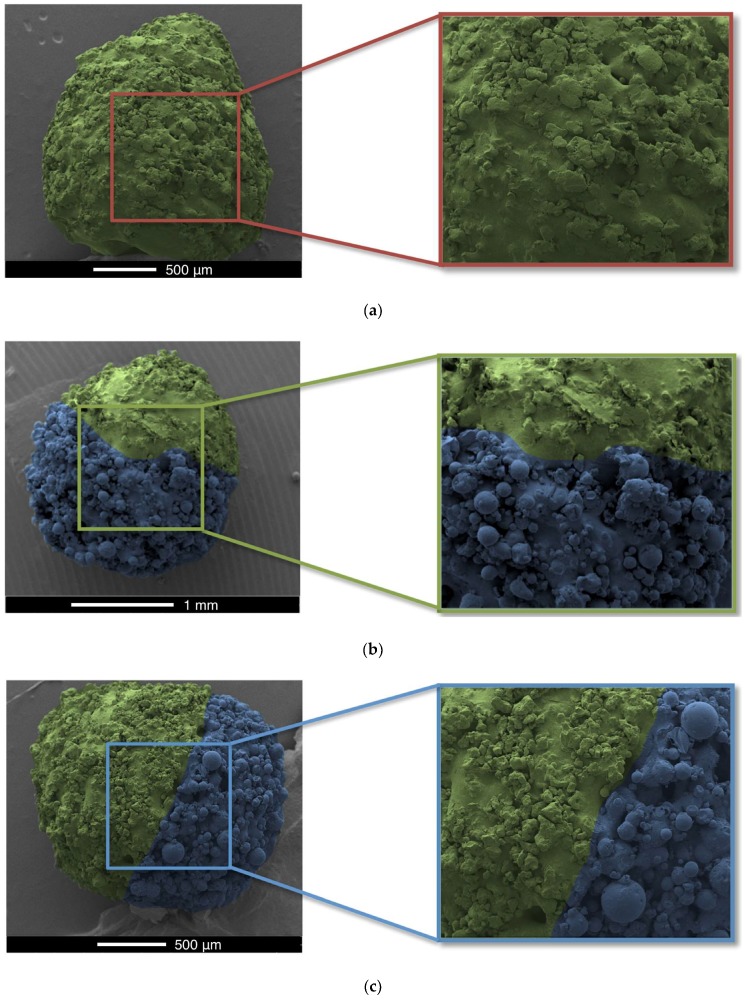
Scanning electron microscopy (SEM) images of the 2 mm (**a**) single miniprintlet, and (**b**) Con A and (**c**) Con B of the dual miniprintlets. The yellow regions represent the EC regions, whereas the blue regions represent the KIR regions.

X-ray micro-CT was used to visualise the 3D structures of the dual miniprintlets (Figure 5). A clear distinction between the two sections can be seen in both configurations, confirming that the two drug regions did not mix during the printing process. The Kollicoat IR regions were denser than the ethyl cellulose regions and are shown in a yellow colour in the images, whereas the ethyl cellulose regions had a dark purple colour. SEM images of the miniprintlets validate the micro-CT results and provide visual confirmation of the differences of the laser effect on the two polymers, even though the same sintering parameters were used (Figure 6). The image of the single miniprintlet shows that ethyl cellulose undergoes a more intense sintering process, where less particles are seen on the surface (Figure 6a). The Con A dual miniprintlets, on the other hand, show two distinct regions, including a molten Ibu/EC region and a Par/KIR sintered region (Figure 6b). As such, it can be concluded that Kollicoat IR undergoes a low intensity sintering process, and thus, a higher space volume between the particles was formed and spherical polymer particles can be distinctively observed on the surface. This can be explained by the difference in the particle shape of each polymer, wherein the Kollicoat IR particles were round, causing them to have less surface area available for contact with nearby particles. On the other hand, the ethyl cellulose particles had irregular, flaky shapes with higher surface area in contact with surrounding particles (Figure 6). The Con B dual miniprintlets also showed two distinct regions, wherein the Ibu/KIR region had bigger round particles indicative of Kollicoat IR and the Par/EC region had irregular particles indicative of paracetamol. 

**Figure 7:**
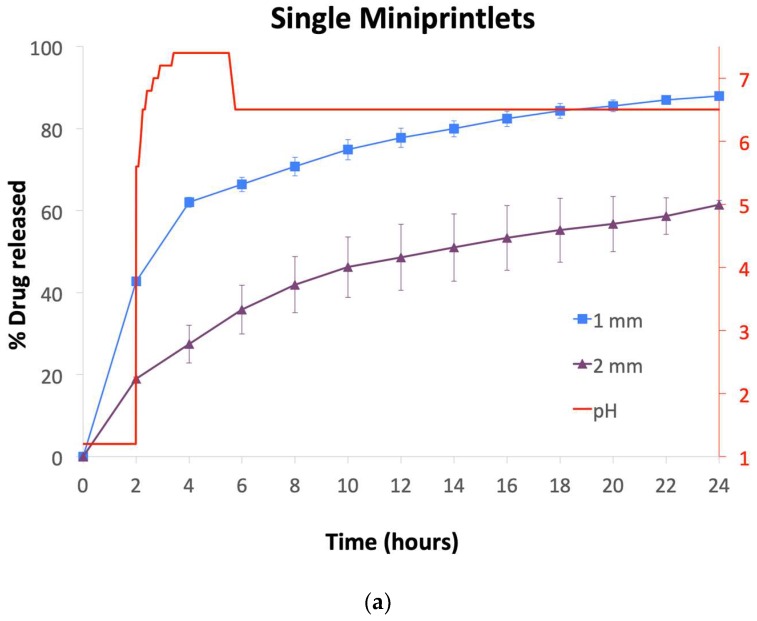

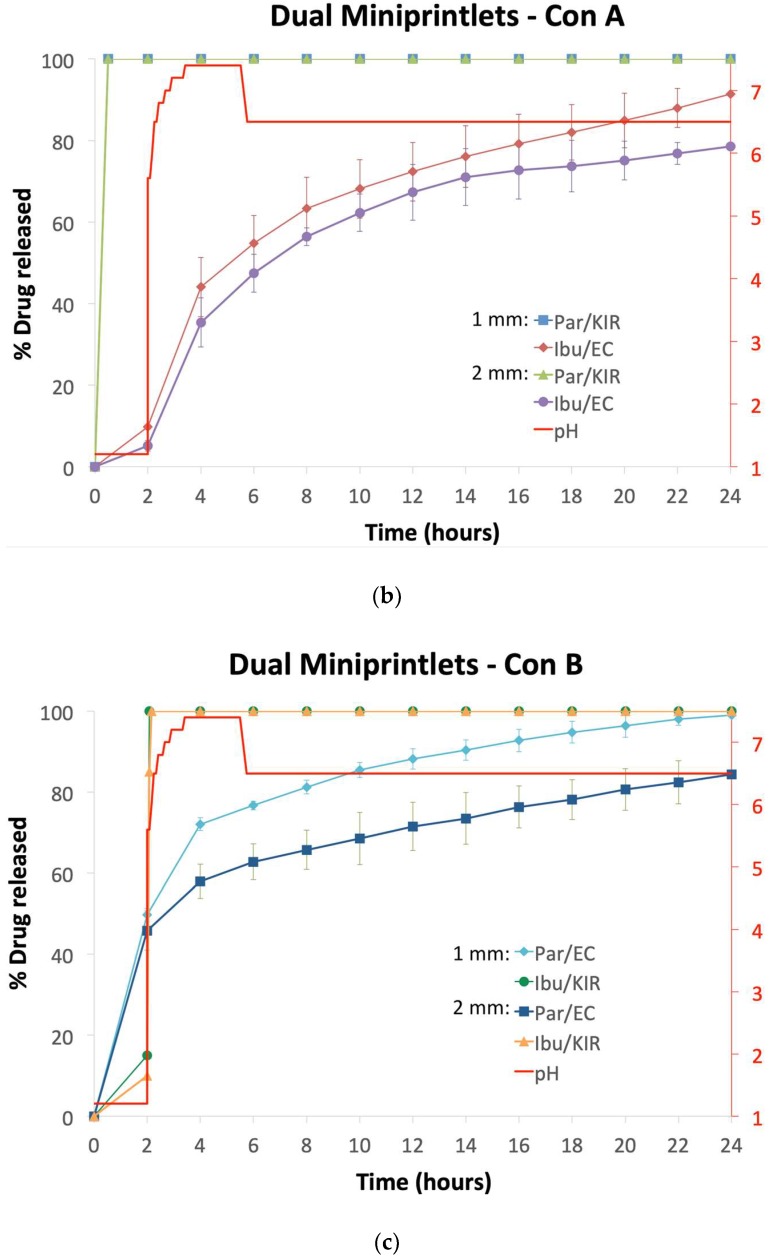
Drug dissolution profiles of the (**a**) single miniprintlets, and (**b**) Con A and (**c**) Con B of the dual miniprintlets. The red line shows the pH values of the media under acidic conditions for 2 h, followed by intestinal conditions using dynamic dissolution apparatus.

Drug dissolution profiles from the miniprintlets were obtained using a dynamic in vitro model, which simulates gastric and intestinal conditions of the gastrointestinal tract (Figure 7) [44]. Despite their larger surface area compared with large monolithic systems, all of the single miniprintlets exhibited sustained and slow paracetamol release, with a drug release of 88% and 61% from the 1 mm and 2 mm miniprintlets, respectively, after 24 h (Figure 7a). As expected, the drug release rate from the miniprintlets was reduced when increasing the diameter. In a previous study, ethyl cellulose has shown very slow release, where only 20% paracetamol was released after 24 h from cylindrical printlets [26]. As such, this shows that the miniprintlets are more suited as a drug delivery platform for SLS printing using ethyl cellulose as the main polymer matrix. Indeed, this can be correlated to the increased surface area resulting from the reduction in diameter of the respective miniprintlets. 

In the dual miniprintlets incorporating two drugs, interestingly, the release profile of paracetamol from the Par/EC region of the 2 mm dual miniprintlets was similar to the 1 mm single miniprintlets, further highlighting that the addition of another region does not affect the original release of the drug and that the two regions maintain their distinct release properties. Regarding Kollicoat IR, owing to the immediate release properties of the polymer, paracetamol was completely released from the Par/KIR region of the Con A dual miniprintlets within 30 min. Additionally, 20% and 15% ibuprofen was released from the Ibu/EC region of the 1 mm and 2 mm Con A dual miniprintlets within the first 2 h, respectively, from the EC layer (Figure 7b). These percentages are probably lower than that of the Par/EC region due to the low solubility of ibuprofen in acidic medium. After being exposed to intestinal conditions (pH 5.6–7.4), acceleration in the dissolution rate was observed, wherein 91% and 79% ibuprofen was released from the 1 mm and 2 mm Con A dual miniprintlets within 24 h, respectively. A similar effect caused by the low drug solubility in acidic medium was seen in the behaviour of Ibu/KIR formulations (Con B), where only 15% and 10% of the drug was released from the 1 mm and 2 mm dual miniprintlets in the first 2 h, respectively (Figure 7c). Once exposed to intestinal conditions, the drug was completely released within 5 min. Par/EC, on the other hand, behaved differently, whereby 50% and 46% was released in the acidic medium from the 1 mm and 2 mm Con B dual miniprintlets, respectively. Under intestinal conditions, a sustained effect was observed, where 99% and 84% of paracetamol was released from the 1 mm and 2 mm Con B dual miniprintlets after 24 h, respectively. 

Conventionally, the production of controlled release multiparticulate systems has been achieved through the use of extrusion-spheronisation and coating [45]. However, this is a multi-step process that is time-consuming and costly, requiring dedicated equipment. Conversely, the SLS technology is a single process that sinters powder particles together using a laser sintering process. As such, the strong coherence between the drug particles and the polymer matrix results in a more sustained release and reduces the amount of drug initially released. Moreover, the use of a slow laser scanning speed increases the duration of the effect of the laser on the powder, allowing more energy to be transmitted, resulting in a higher degree of sintering and consequently sustaining the drug release [46,47]. Unlike coating, damage to the surface of the miniprintlets does not affect their release properties, as the drugs are uniformly dispersed in a matrix structure and are not covered by a protective layer. Moreover, in the case of coating, the drug release becomes generally fast once the coating ruptures or dissolves, whereas in this matrix system, the drug release remains constant [48]. This can be explained by the insoluble nature of ethyl cellulose, whereby the drug molecules diffuse through the matrix. Compared with a single-unit dosage form, risks of dose-dumping and peak plasma fluctuations are minimised with this multiparticulate system, as each miniprintlet acts as a separate drug depot, having an individualised drug dose and release mechanism [49]. In particular, this could benefit narrow therapeutic index drugs, which are characterised by having a small difference between the therapeutic and toxic doses, wherein improper dosing could lead to undesirable adverse effects or inefficient therapy [50].

The use of dual miniprintlets offers the choice of combining multiple APIs, where each drug could have a different release profile within the same miniprintlet. The formulation of the miniprintlets in a combination of instant and extended release forms could offer benefits, such as convenient dosing with a lower frequency of intake [51], while providing longer lasting analgesic and antipyretic effects. As such, drug combinations with different doses, having the same ratio of APIs, could be prepared to suit the need of patients from different age groups [52,53]. The development of a patient-centric platform could be particularly beneficial for paediatric and geriatric patients, where dose adjustments are needed due to variations in pharmacodynamic and pharmacokinetic characteristics [54]. In contrast to previously proposed pathways for personalised medications, the use of the miniprintlet platform is much simpler and more efficient, as it does not require altering of the shape or dimensions of the dosage form, both of which could affect the drug release [55,56]. The use of this novel approach could enhance the treatment regime, moving it away from a ‘one size fits all’ approach toward personalised medicines, which are safer and more effective [57]. 

## 4. Conclusions

In this work, we demonstrated that miniprintlets prepared using SLS 3DP offer a novel drug delivery approach with high flexibility and control over the drug content and release properties. Fine-tuning of the therapeutic effect can be achieved by modulating parameters such as the dimensions and matrix composition, which in turn can be leveraged to produce multi-drug systems. To our knowledge, this study is the first to demonstrate the possibility of combining two rate-controlling systems in such small and intricate pharmaceutical dosage forms, enabling the individual programming of each drug. As such, this emphasises the value of this technology, making it favourable over other commercial fabrication systems for the production of pharmaceuticals. 

## Figures and Tables

**Table 1:** Compositions of the single and dual miniprintlets.

Miniprintlets *	Paracetamol (Par)	Ibuprofen (Ibu)	Kollicoat Instant Release (KIR)	Ethyl Cellulose (EC)
Single	5%	-	-	92%
Dual–Con A				
Par/KIR	6.5%	-	56.5%	-
Ibu/EC	-	3.5%	-	30.5%
Dual–Con B				
Ibu/KIR	-	3.5%	30.5%	-
Par/EC	6.5%		-	56.5%

* All formulations contain 3% *w*/*w* Candurin^®^ Gold Sheen.

**Table 2:** Characteristics of the miniprintlets (SD—standard deviation).

Miniprintlets	Weight (mg ± SD)	Diameter (mm ± SD)	Paracetamol Content (% ± SD)	Ibuprofen Content (% ± SD)
Single				
1 mm	0.84 ± 0.03	1.14 ± 0.06	101.1 ± 0.5	-
2 mm	3.90 ± 0.13	1.99 ± 0.06	96.9 ± 0.2	-
Dual–Con A				
1 mm	0.67 ± 0.03	1.04 ± 0.02	100.1 ± 1.5 *	99.4 ± 1.0 *
2 mm	4.27 ± 0.15	2.03 ± 0.02	98.5 ± 1.5 *	99.6 ± 1.4 *
Dual–Con B				
1 mm	0.50 ± 0.02	1.01 ± 0.04	99.2 ± 0.2 *	98.3 ± 1.1 *
2 mm	4.10 ± 0.08	2.00 ± 0.03	96.6 ± 0.5 *	100.2 ± 1.5 *

* The following values were calculated by printing single miniprintlets with compositions identical to the corresponding region being tested.
